# Left ventricular remodeling following septal myectomy in hypertrophic obstructive cardiomyopathy

**DOI:** 10.1016/j.xjon.2022.05.018

**Published:** 2022-06-27

**Authors:** Tsuyoshi Yamabe, Jonathan Ginns, Vijay Vedula, Jay S. Leb, Yuichi J. Shimada, Shepard D. Weiner, Hiroo Takayama

**Affiliations:** a Division of Cardiothoracic and Vascular Surgery, New York Presbyterian Hospital, Columbia University Medical Center, New York, NY; b Department of Cardiovascular Surgery, Shonan-Kamakura General Hospital, Kamakura, Kanagawa, Japan; c Department of Cardiology, Heart Hospital of Austin, Tex; d Department of Mechanical Engineering, Columbia University, New York, NY; e Department of Radiology, New York Presbyterian Hospital, Columbia University Medical Center, New York, NY; f Department of Medicine Cardiology, New York Presbyterian Hospital, Columbia University Medical Center, New York, NY

**Keywords:** hypertrophic cardiomyopathy, left ventricular outflow tract obstruction, septal myectomy, left ventricular myocardium remodeling, 3-dimensional computed tomography, systolic anterior motion, mitral regurgitation, 3D-CT, 3-dimensional computed tomography, AF, atrial fibrillation, CTA, computed tomography angiogram, ECG, electrocardiogram, HCM, hypertrophic cardiomyopathy, LV, left ventricle, LVOT, left ventricular outflow tract, NYHA, New York Heart Association, SAM, systolic anterior motion, SM, septal myectomy, TEE, transesophageal echocardiography, TTE, transthoracic echocardiography, VM, virtual myectomy

## Abstract

**Objectives:**

The purpose of this study is to determine whether or not left ventricular remodeling can be induced after septal myectomy in patients with obstructive hypertrophic cardiomyopathy, and if so, how it occurs, using gated cardiac computed tomography.

**Methods:**

Fifty patients with hypertrophic obstructive cardiomyopathy who underwent septal myectomy along the septal band between March 2016 and July 2020 were retrospectively reviewed. Recent consecutive 19 patients underwent postoperative cardiac computed tomography. In these patients, volumes of the septal band and thickness of 17 left ventricular myocardial segments were measured to determine the changes after surgery.

**Results:**

The resection volume predicted by preoperative computed tomography and the actual resection volume were 6.7 ± 3.3 mL and 6.4 ± 2.7 mL. In-hospital mortality was 0%. Moderate or greater mitral valve regurgitation and systolic anterior motion decreased from 56% to 6% and 86% to 6%, respectively. Median preoperative ventricular septal thickness and left ventricular outflow tract pressure gradient at rest decreased from 20.0 mm (interquartile range, 17.0-24.0 mm) and 74.0 mm Hg (interquartile range, 42.5-92.5 mm Hg) to 14.0 mm (interquartile range, 11.5-16.0 mm) and 15.5 mm Hg (interquartile range, 12.1-21.5 mm Hg), respectively. Postoperative computed tomography confirmed a reduction in septal band volume of 5.7 ± 2.8 mL. Total left ventricular myocardial volume was reduced by 12.9 ± 8.8 mL, which exceeded the volume reduction of the resected septal band. All segments except the basal inferior and basal inferolateral regions showed a significant decrease in wall thickness by a median of 6.4%.

**Conclusions:**

Properly performed septal myectomy may induce remodeling of the entire left ventricle, not just the resected area.

## Graphical abstract

Gated cardiac computed tomography angiography showed that whole left ventricle remodeling is induced after septal myectomy in patients with hypertrophic obstructive cardiomyopathy.

**Figure:**
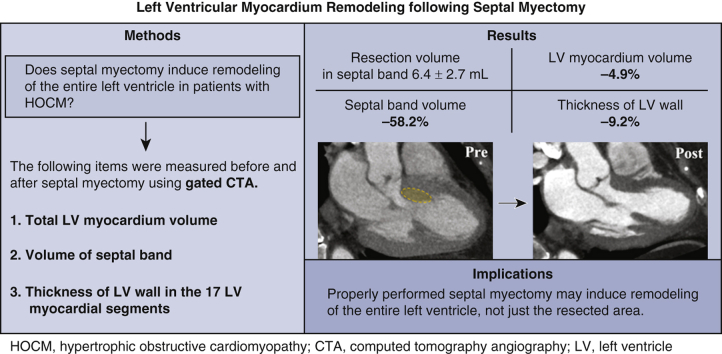

**Figure:**
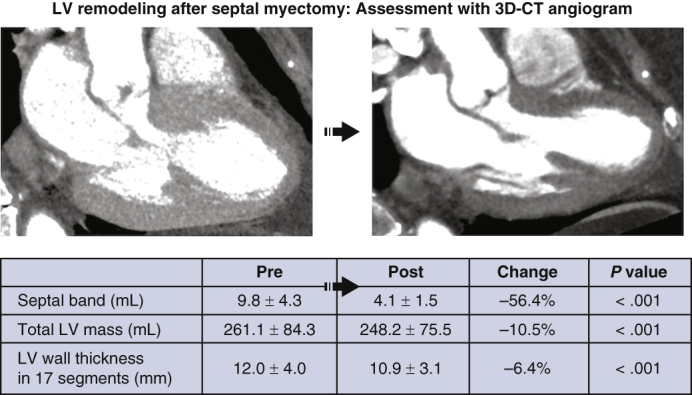
The LVOT obstruction has been removed and the LV myocardium remodeling has occurred.

**Central Message**

Properly performed septal myectomy may induce remodeling of the entire left ventricle beyond the actual resection area.

**Perspective**

Septal myectomy has the potential to reverse the progression of left ventricular hypertrophy.

Up to 70% of patients with hypertrophic cardiomyopathy (HCM) have the left ventricular outflow tract (LVOT) obstruction.^1^ The left ventricle (LV) develops diffuse hypertrophy due to a combination of 2 factors: a disease process in which genetic factors affect cardiomyocytes, and secondary hypertrophy caused by increased afterload.^2^ Septal reduction therapy with surgical septal myectomy (SM) and alcohol septal ablation is utilized to relieve symptoms refractory to maximal medical therapy.^3^, ^4^, ^5^ These procedures reduce the LVOT pressure gradient and alleviate the progressive heart failure in patients with obstructive HCM.^6^, ^7^, ^8^

However, it has been demonstrated that there is no direct relationship between symptoms of heart failure and the degree of LV hypertrophy,^9^ and little is known about how and which part of the LV myocardium is affected by the reduction in LVOT pressure gradient after septal reduction therapy. Considering that the magnitude of LV hypertrophy is associated with sudden cardiac death and an important predictor of outcome in patients with HCM,^10^^,^^11^ changes in LV myocardial mass may have an implication in the prognosis.

In an effort to make SM more reproducible, we have introduced 2 surgical concepts: septal band and virtual myectomy (VM).^12^ The septal band is a characteristic hypertrophic septal structure seen in HCM with basal LVOT obstruction, which extends from beneath the left coronary annulus, running obliquely in a clockwise fashion along the septal wall down to the base of the posteromedial papillary muscle. Resecting this band ameliorates the obstruction. VM is a preoperative assessment tool to objectively measure the extent and volume of the myocardial resection using 3-dimensional computed tomography (3D-CT). Use of 3D-CT provided us with a unique opportunity to study LV remodeling following SM.

In the present study, we hypothesized that SM would lead to remodeling of the LV myocardium beyond the resection area, and evaluated the changes of the LV myocardial volume in detail following SM.

## Methods

### Study Design and Patient Selection

This is a single-center retrospective study approved by Columbia University Medical Center Institutional Review Board. Obtaining informed consent was waived because of the retrospective nature of this study (IRB No.: AAAT2539, first approval date: August 11, 2020, and most recent approval date: November 16, 2020).

Fifty patients with obstructive HCM who underwent SM at our institution between March 2016 and July 2020 were retrospectively reviewed and analyzed. Preoperative demographic characteristics, CT and echocardiogram (ECG) data, procedure-related details, and postoperative complications (following the definition of the Society of Thoracic Surgery Adult Cardiac Database version 2.9 whenever available) were collected by reviewing electronic medical records. Recent 19 consecutive patients underwent postoperative ECG-gated cardiac CT angiogram (CTA) in addition to preoperative CTA. In these patients, volumes of the septal band and thickness of the 17 LV myocardial segments, as recommended by the American Heart Association Standardized Myocardial Segmentation and Nomenclature for tomographic imaging guideline,^13^ were measured to determine the changes following SM. Postoperative ECG-gated cardiac CTA was done at a median of 2.5 months (interquartile range [IQR], 1.8-3.4 months) after surgery. Specifically, 3 patients underwent postoperative CTA within 1 month, 11 patients between 1 and 3 months, 3 patients between 3 and 6 months, and 2 patients between 6 months and 1 year after surgery.

### Patient Management

#### ECG

All patients underwent a comprehensive preoperative transthoracic echocardiogram (TTE), with Valsalva maneuver. LVOT peak velocity was measured by continuous wave Doppler echocardiography; if the LVOT peak pressure gradient exceeded 100 mm Hg, no provocative maneuver was performed. The degree of mitral regurgitation was assessed on a scale of 0 to 4 (0 = none, 1 = trivial, 2 = mild, 3 = moderate, and 4 = severe). All patients underwent predischarge and follow-up TTE. The follow-up TTE was performed in 6 patients within 1 month, in 11 patients between 1 and 3 months, in 7 patients between 3 and 6 months, in 10 patients between 6 months and 1 year, and in 15 patients after 1 year postoperatively. Transesophageal echocardiography (TEE) was performed in the operating room, where LVOT pressure gradient after SM was measured with premature ventricular contraction provocation.

#### Gated 3D-CTA

The details of cardiac 3D-CT and VM have been described previously.^12^ In brief, a 320-detector low volumetric scanner (Aquilion One; Toshiba America Medical System) was used to perform a 350 msec continuous ECG-gated volume scan of the heart, focusing on the late diastolic phase (90% of the R-R interval). The resulting images were postprocessed with Vitrea software (Vital Images). 3D reconstruction was performed to visualize areas of LV hypertrophy and surrounding structures associated with relevant surgical anatomies. The volumes of total LV myocardium and septal band were calculated. In addition, the thickness in 17 segments of LV myocardium, including the ventricular septum were measured.

#### Surgical procedure

**Figure E1:**
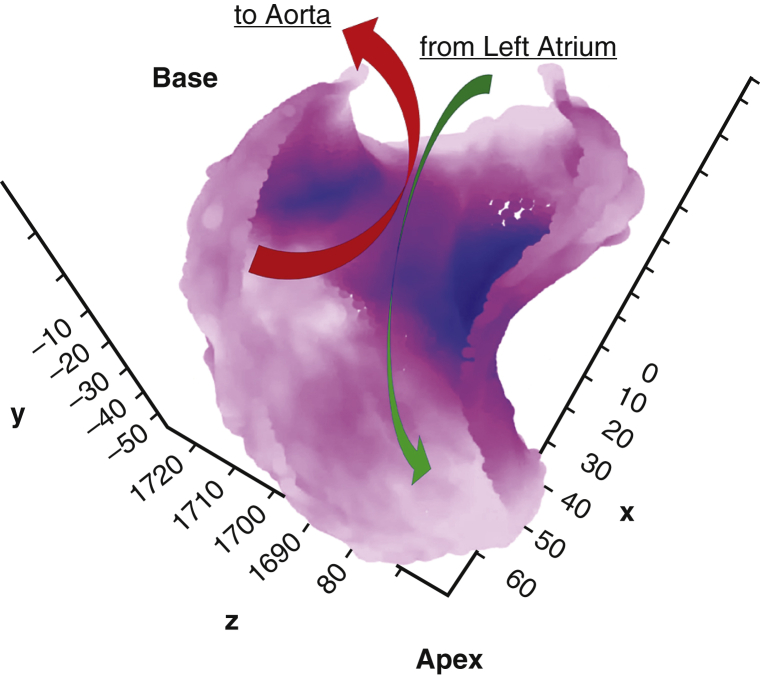
The upper part of the figure is the base, where the left ventricular outflow tract and mitral valve annulus are located, and the lower part is the apex. Septal band is localized as a hypertrophic septal structure (*darkest blue*), which starts at the basal anterior wall and runs clockwise fashion when viewed from the apex, and generally continues to the mid inferior wall.

**Figure E2:**
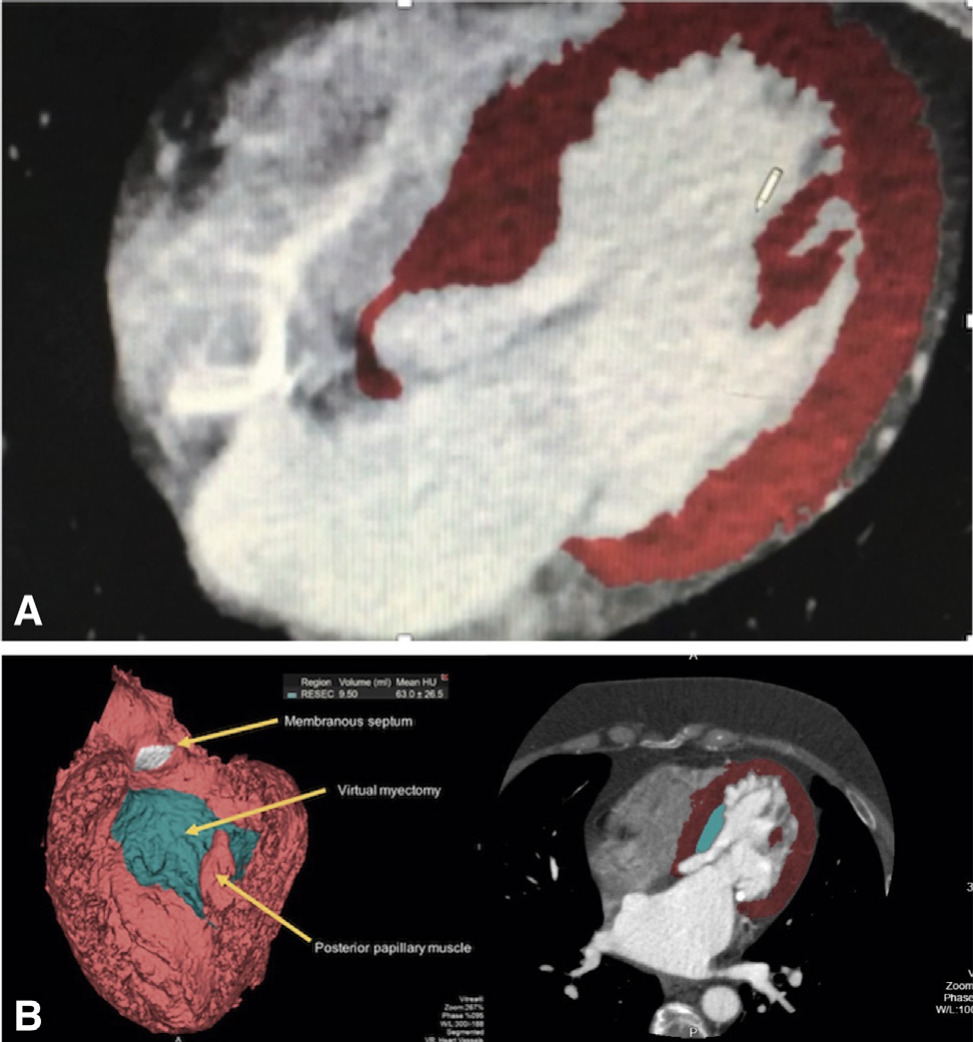
A, Longitudinal section of the left ventricle. Left ventricular myocardium is identified and segmented in a long axis image of computed tomography. B, Planned resection area in a 3-dimensional reconstruction image. The portion of septal band from the left trigone to the posteromedial papillary muscle (*green*) is the target resection area. The resection volume, extent, and thickness are rehearsed in the virtual myectomy. The membranous septum is indicated by a gray area. At present, the measurement of muscle segment volume was done by a person. To assess the interobserver variability, the measurements were performed by 2 blinded physicians who received a special training for the measurement and repeated a couple of times to actually measure in front of 1 expert and have him teach them the tips. The interclass correlation coefficient (2, 1) was 0.96 (95% CI, 0.93-0.98). Bland-Altman plots for the variability are shown in Figure E3. This graph suggested the presence of proportional error, where the difference in measurements increases in proportion to the amount of resection measured by virtual myectomy.

SM was recommended based on the contemporary HCM guidelines.^14^^,^^15^ We have introduced 2 concepts toward making SM more objective: septal band (Figure E1) and VM (Figure E2).^12^ Although not an accepted anatomical concept, the septal band is a characteristic hypertrophic continuous septal structure seen in HCM with basal LVOT obstruction, which starts basally beneath the left coronary annulus, extends obliquely in a clockwise fashion (viewed from the apex) along the septal wall down to the base of the posteromedial papillary muscle. With VM, the area and depth of the hypertrophic myocardium to be resected, which typically follows the septal band, were determined by our HCM cardiologists and surgeons, and the resection volume was calculated. The detail of our SM was previously described.^12^^,^^16^ To summarize, we enforce SM with the following 3 steps: resection of tissue via longitudinal parallel-guided incisions in the septum as described in the original Morrow procedure with extension toward the LV apex (Morrow part), left-sided excision toward the left trigone (left lateral part), then right-sided excision extending down to the base of the posterolateral papillary muscle (right lateral part). During SM, the septal band was resected to achieve the VM-predicted resection volume.

Mitral valve intervention was added whenever deemed appropriate, typically for abnormal secondary chordae or papillary muscles inserting to the anterior mitral leaflet. Patients with preoperative atrial fibrillation (AF) underwent surgical ablation procedure with pulmonary vein isolation and left atrial appendage closure. Anatomical assessment of the LVOT and pressure measurement were performed before and after cardiopulmonary bypass under TEE guidance to confirm the appropriateness of SM.

### Statistical Analysis

All statistical analyses were performed with R version 4.0.0 (R Foundation for Statistical Computing). Continuous variables were tested for normality using the Kolmogorov-Smirnov test. Nonnormality variables were expressed as median (IQR), whereas normally distributed variables were mean ± SD. Categorical data were described with numbers and percentages of the total. All statistical test values were 2-sided.

The Friedman test was used to compare echocardiography data at 4 time points, and the post hoc Bonferroni correction was applied to assess differences in variables in 6 pairwise comparisons using the Wilcoxon signed rank test. To declare statistical significance between these 4 time points, 6 different multiple comparisons must be made, so the *P* value must be less than .0083 (.05 divided by 6). The difference in incidence of septal anterior motion was analyzed using the Cochran *Q* test. A linear model was run to compare the pre- and postoperative data for CTA. These data were measured from the same patient at 2 different time points and are necessarily linked. Therefore, the paired *t* test was driven to compare the mean values between the corresponding patients. The Pearson product moment correlation coefficient was also used to obtain the *r* value and associated *P* value.

**Figure E3:**
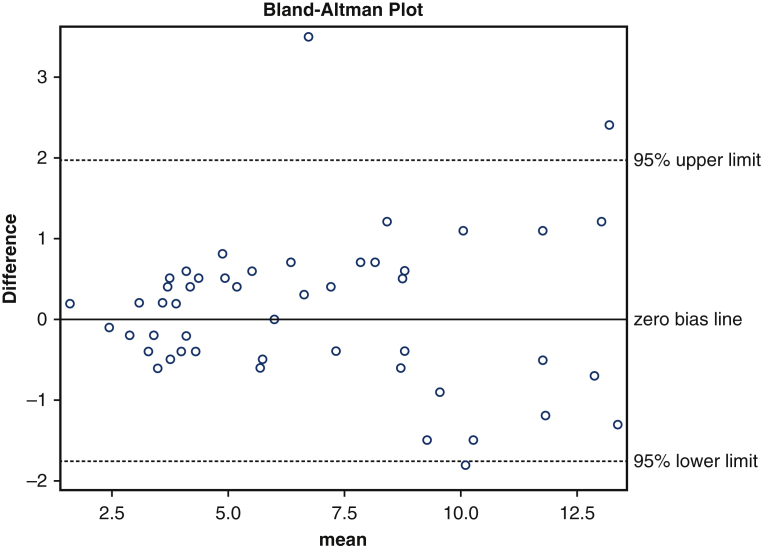
Bland-Altman plots of interobserver variability. The mean values of pairs of measurements are plotted against the difference between the measurements. The *black continuous line* represents the arithmetic mean and the *black dotted lines* represent 95% limits of agreement.

To evaluate the interobserver bias, VM was blindly performed by 2 physicians (Y.T. and K.A.), and the Bland-Altman plot was created to examine whether systematic errors were introduced into the measurements (Figure E3).

## Results

### Patient Characteristics

The baseline characteristics of the patients are listed in Table 1. The median age was 58.0 years (IQR, 48.3-64.0 years) with 50% women. Diabetes was seen in 14% and AF in 24%. Syncopal episode occurred in 16% of patients. One patient presented with infective endocarditis involving both the aortic and mitral valves. Preoperative New York Heart Association (NYHA) functional class 3 or greater was observed in 56% of patients with the mean preoperative NYHA functional class of 2.5 ± 0.8. The echocardiographic results are summarized in Table 2. The LV end-diastolic diameter was 43.9 ± 5.4 mm with LV ejection fraction of 66.1% ± 4.9%. LVOT pressure gradient at rest was 74.0 mm Hg (IQR, 42.5-92.5 mm Hg), and ventricular septal thickness was 20.0 mm (IQR, 17.0-24.0 mm). Systolic anterior motion (SAM) was found in 86% of patients, and moderate or greater mitral regurgitation in 56%. The indications for myectomy in patients without SAM were midventricular type in 3 patients, HCM with severe LVOT obstruction complicated by infective endocarditis in 1 patient, and HCM with severe LVOT obstruction in 2 patients. None of the patients we analyzed had apical type.

**Table 1:** Patient characteristics

Variable	Septal myectomy (N = 50)
Age (y)	58.0 (48.3-64.0)
Female sex	25 (50.3)
Body surface area (m^2^)	2.0 (1.9-2.2)
Hypertension	33 (66.0)
Dyslipidemia	21 (42.0)
Diabetes	7 (14.0)
Hb	13.5 (11.9-14.3)
Htc	40.0 (36.7-43.2)
CAD	6 (12.0)
CVD	1 (2.0)
COPD	3 (6.0)
PAD	1 (2.0)
CKD	6 (12.0)
AF	12 (24.0)
Infective endocarditis	1 (2.0)
NYHA functional class	
1	6 (12.0)
2	17 (34.0)
3	25 (50.0)
4	2 (4.0)

Values are presented as median (interquartile range) or as n (%). *Hb*, Hemoglobin; *Htc*, hematocrit; *CAD*, coronary artery disease; *CVD*, cerebrovascular disease; *COPD*, chronic obstructive pulmonary disease; *PAD*, peripheral artery disease; *CKD*, chronic kidney disease; *AF*, atrial fibrillation; *NYHA*, New York Heart Association.

**Table 2:** Temporal changes in echocardiography results

Variable	Preoperative (n = 50)	Intraoperative (n = 50)	Discharge (n = 50)	Follow-up (n = 50)	*P* value
LVEF (%)	66.1 ± 4.9	–	61.7 ± 6.4	61.1 ± 7.0	<.001∗†
LVEDD (mm)	43.9 ± 5.4	–	44.7 ± 6.1	45.0 ± 5.9	.478‡
LVESD (mm)	25.2 ± 4.8	–	28.5 ± 5.9	28.8 ± 5.8	.148‡
Peak LVOT PG (mm Hg)	74.0 (42.5-92.5)	10.0 (8.0-12.0)	22.5 (15.0-34.5)	15.5 (12.1-21.5)	<.001∗†§‖¶
Ventricular septal thickness (mm)	20.0 (17.0-24.0)	14.0 (13.0-15.0)	14.0 (13.0-15.5)	14.0 (11.5-16.0)	<.001∗†§
Mitral regurgitation					
None	2 (4.0)	16 (32.0)	12 (24.0)	22 (44.0)	<.001∗†§
Trace	5 (10.0)	8 (16.0)	16 (32.0)	13 (26.0)	
Mild	15 (30.0)	23 (46.0)	19 (38.0)	12 (24.0)	
Moderate	15 (30.0)	3 (6.0)	3 (6.0)	3 (6.0)	
Severe	13 (26.0)	0	0	0	
SAM	43 (86.0)	3 (6.0)	2 (4.0)	2 (4.0)	<.001

Values are presented as mean ± SD, median (interquartile range), or n (%). *LVEF*, Left ventricular ejection fraction; *LVEDD*, left ventricular end-diastolic diameter; *LVESD*, left ventricular end-systolic diameter; *LVOT*, left ventricular outflow tract; *PG*, pressure gradient; *SAM*, systolic anterior motion.

∗ Significantly different between preoperative and discharge.

† Significantly different between preoperative and follow-up.

‡ No difference in all the comparisons of the 4 groups.

§ Significantly different between preoperative and intraoperative.

‖ Significantly different between intraoperative and discharge.

¶ Significantly different between intraoperative and follow-up.

### Operative Details

Intraoperative data are described in Table 3. The mean myocardial resection volume predicted by VM was 6.7 ± 3.3 mL, and the actual resection volume was 6.4 ± 2.7 mL (*r* = 0.72; *P* < .001). Isolated SM was performed in 36% (18 out of 50) of patients. Mitral valve intervention was performed in 56% (28 out of 50) of patients, of whom 23 underwent mitral valvuloplasty and 5 underwent mitral valve replacement. Mitral valvuloplasty consisted of abnormal chord resection in 21 cases, papillary muscle realignment in 2 cases, and leaflet plication in 4 cases. Mitral valve replacement was performed for infective endocarditis (n = 1), extensive fibrosis of the valve leaflets (n = 1), bileaflet tethering due to chordae shortening (n = 1), and excessive thickening and foreshortening of the anterior leaflet (n = 2). The aortic valve procedure included 2 valve repairs for aortic insufficiency, and 2 valve replacements for aortic stenosis or infective endocarditis. All 12 patients with preoperative AF had the surgical ablation with pulmonary vein isolation and left atrial appendage ligation. The cardiopulmonary bypass and aortic crossclamp times were 124.5 min (IQR, 110.0-146.3 min) and 89.0 min (79.0-105.0 min), respectively.

**Table 3:** Operative details

Variable	Septal myectomy (N = 50)
Isolated septal myectomy	18 (36.0)
Concomitant procedures	
Aortic valve procedure	4 (8.0)
Mitral valve repair	23 (46.0)
Abnormal chord resection	21 (42.0)
Papillary muscle realignment	2 (4.0)
Leaflet plication	4 (8.0)
Mitral valve replacement	5 (10.0)
CABG	1 (2.0)
Maze	12 (24.0)
LAA close	21 (42.0)
CPB time (min)	124.5 (110.0-146.3)
ACC time (min)	89.0 (79.0-105.0)
Predicted resection volume∗ (mL)	6.7 ± 3.3
Resection volume† (mL)	6.4 ± 2.7
VSD	0

Values are presented as mean ± SD, median (interquartile range), or n (%). *CABG*, Coronary artery bypass graft; *LAA*, left atrial appendage; *CPB*, cardiopulmonary bypass; *ACC*, aortic crossclamp; *VSD*, ventricular septal defect.

∗ Virtual myectomy.

† Virtual myectomy-guided septal myectomy.

TEE performed in the operating room after SM showed that the septal thickness decreased to 14.0 mm (IQR, 13.0-15.0 mm) and the LVOT pressure gradient improved to 10.0 mm Hg (IQR, 8.0-12.0 mm Hg). Moderate mitral regurgitation and SAM remained in 6% (3 out of 50) of patients. No postoperative ventricular septal defect was observed.

### Postoperative Outcomes

There was no in-hospital mortality and stroke. One patient had mediastinal re-exploration for bleeding. Complete atrioventricular block requiring permanent pacemaker implantation was required in 2 patients. Sinus rhythm was achieved in 58.3% (7 out of 12) of the patients who had undergone surgical ablation. The mean NYHA functional class at follow-up was 1.2 ± 0.4.

### Temporal Changes in ECG Parameters

The postmyectomy septal thickness did not change over time. The LVOT pressure gradient mildly increased at discharge (22.5 mm Hg [IQR, 15.0-34.5 mm Hg] from 10.0 mm Hg [IQR, 8.0-12.0 mm Hg] at intraoperative TEE), and then decreased again at follow-up (15.5 mm Hg [12.1-21.5 mm Hg]). One of 3 patients with postoperative moderate residual mitral regurgitation and SAM had an improvement in SAM, and the other 2 patients remained unchanged.

### Changes in the LV Myocardium

**Figure 1:**
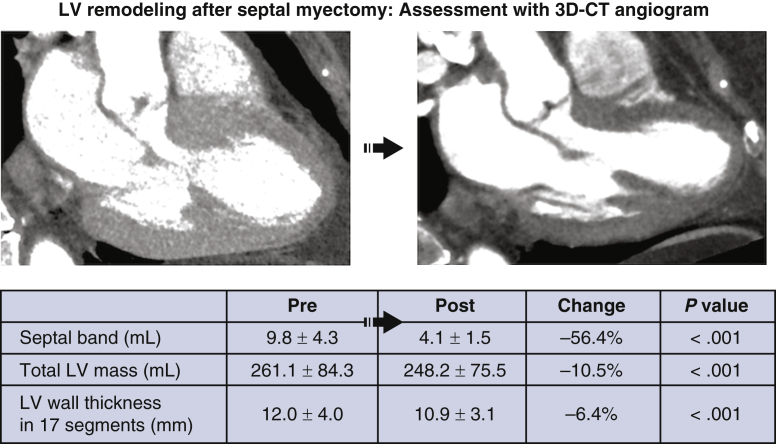
Left ventricle (*LV*) remodeling after septal myectomy: Assessment with 3-dimensional computed tomography (*3D-CT*) angiogram. The LV outflow tract obstruction has been removed and LV myocardium remodeling has occurred.

**Figure 2:**
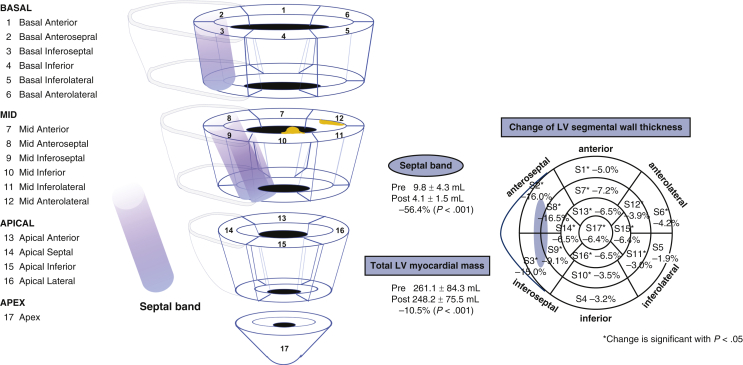
The 17 segments of left ventricle (*LV*) myocardium. Schematic representation of the 17 LV segments. Significant decreases in the LV myocardial mass in all segments except the basal inferior and basal inferolateral regions. Especially, decrease >15% was observed in the regions mainly on the septal band.

Postoperative cardiac 3D-CTA showed that the septal band volume decreased from 9.8 ± 4.3 mL preoperatively to 4.1 ± 1.5 mL postoperatively, corresponding to the resection volume (*r* = 0.82; *P* < .001). On the other hand, the volume of the total LV myocardium decreased from 261.1 ± 84.3 mL to 248.2 ± 75.5 mL, a difference of 12.9 mL, which is beyond the actual resection volume (Figure 1). The changes in the wall thicknesses in 17 segments of LV myocardium (measured at end-diastole) are demonstrated in Figure 2. The mean change was 1.1 mm (–6.4%). The wall thickness significantly decreased in all segments except the basal inferior and basal inferolateral regions. In particular, a decrease >15% was observed in the regions mainly on the septal band, including the basal anteroseptal, basal inferoseptal, and mid-anteroseptal areas (Table 4).

**Table 4:** Changes in segmental left ventricle (LV) wall thickness following virtual myectomy (VM)-guided septal myectomy (SM)

LV segment		Pre-SM (mm) (n = 19)	Post-SM (mm) (n = 19)	% Change (n = 19)	*P* value
Basal					
1	Anterior	12.5 ± 5.4	11.5 ± 3.7	–5.0	.04
2	Anteroseptal	19.1 ± 3.6	16.6 ± 4.3	–16.0	<.001
3	Inferoseptal	13.5 ± 4.2	11.9 ± 5.0	–15.0	.003
4	Inferior	10.5 ± 1.8	10.3 ± 1.8	–3.2	.07
5	Inferolateral	10.3 ± 1.2	10.2 ± 1.4	–1.9	.219
6	Anterolateral	12.0 ± 2.6	11.5 ± 2.0	–4.2	.017
Mid					
7	Anterior	11.2 ± 2.2	10.4 ± 2.1	–7.2	.002
8	Anteroseptal	17.3 ± 4.4	14.4 ± 2.9	–16.5	<.001
9	Inferoseptal	13.7 ± 4.4	12.6 ± 3.7	–9.1	.039
10	Inferior	10.6 ± 1.7	10.4 ± 1.8	–3.5	.004
11	Inferolateral	10.3 ± 1.3	10.2 ± 1.5	–3.0	.03
12	Anterolateral	11.0 ± 2.4	10.7 ± 2.2	–3.9	.009
Apical					
13	Anterior	11.2 ± 3.5	10.6 ± 3.0	–6.5	.001
14	Septal	11.4 ± 3.4	10.9 ± 3.3	–6.5	.028
15	Inferior	9.8 ± 3.0	9.2 ± 2.4	–6.4	.005
16	Lateral	10.0 ± 2.8	9.4 ± 2.4	–6.5	.005
Apex					
17	Apex	9.8 ± 3.0	9.2 ± 2.5	–6.4	.025

## Discussion

**Figure 3:**
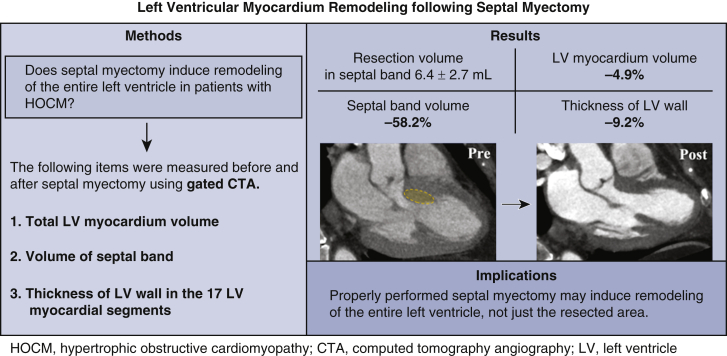
Gated cardiac computed tomography angiography (*CTA*) showed that whole left ventricle (*LV*) remodeling is induced after septal myectomy in patients with hypertrophic obstructive cardiomyopathy (*HOCM*).

In this study, we investigated the subsequent changes in the LV myocardium following SM. The main finding was that the LV myocardium after SM decreased more than the actual amount of resection, with significant reduces in almost all LV wall segments Figure 3.

3D-CTA allowed us to further study LV remodeling following SM. Most notably, the overall reduction in LV myocardial mass was significantly greater than the actual resection volume. The LV wall was divided into 17 sections to investigate the reginal changes in myocardial thickness. The reduction in LV wall thickness was observed in all segments albeit with heterogeneous extents. Using ECG, Deb and colleagues^17^ measured LV mass and LV mass index preoperatively and postdischarge in patients who underwent SM for obstructive HCM, and reported that significant decreases in LV mass and LV mass index occurred early postoperatively. Similarly, Nguyen and colleagues^18^ described that LV mass index decreased during the early postoperative period after septal resection. A small case series with cardiac magnetic resonance imaging reported similar findings.^19^ Finally, Tang and colleagues^20^ showed that SM may lead to relative increase in the LV mass with late gadolinium enhancement; however, their data could be interpreted as unchanged fibrotic mass in the presence of regressed viable myocardial mass.^21^ Together, these data suggest significant contribution of afterload from LVOT obstruction to the LV hypertrophy in HCM. If this is the case, SM in the early stages of HCM, when there is still viable myocardium that can be remodeled before fibrosis progresses, is recommended. In fact, time from diagnosis to surgery has been identified as an independent factor in postoperative disease progression, including new onset of AF, NYHA functional class worsening, reintervention, and death, and the influence of taking more than 5 years to treat, even if successful in alleviating symptoms and LVOT gradients, is significant, with a 3.4-fold increase in risk of these diseases compared with treating <3 years.^22^ In addition to symptomatic improvement, SM has been reported to be associated with a number of clinical benefits: when SM relieves LV pressure loading, it also improves myocardial microcirculation and resolves ischemia.^23^ Furthermore, improvement in diastolic dysfunction combined with improvement in mitral regurgitation may resolve the pressure and volume load on the left atrium, and prevent new AF and secondary pulmonary hypertension, resulting in improved survival.^24^ On the other hand, despite successful SM, a number of patients die from cardiovascular causes in the long-term. In surgically untreated patients with HCM, a correlation has been identified between LV wall thickness and the risk of sudden death, progression to heart failure, and all-cause mortality.^10^ Given that the degree of LV hypertrophy is closely associated with the occurrence of fatal arrhythmias and sudden death, although it is possible that LV remodeling by SM itself may be directly related to improved survival, it has been reported that the degree of postoperative myocardial hypertrophy was not associated with these adverse events,^9^^,^^25^ and the mechanism remains to be elucidated.

Further observation is needed to clarify the future course of these LV changes and their influence on a patient's prognosis.

### Limitations

This study was a single-center retrospective study with a small sample size, and thus the findings may not be generalizable. Furthermore, the timing of the postoperative CT was not consistent among patients. The small cohort size did not allow further adjustment upon analysis.

## Conclusions

The present study showed that SM appears to induce remodeling of the entire LV myocardium, not just the resected area.

### Webcast

You can watch a Webcast of this AATS meeting presentation by going to: https://aats.blob.core.windows.net/media/Publications/AM21_A05%20-%20HOCM%20and%20LV%20Remodeling.mp4-PresPlusDisc.mp4.

**Figure:**
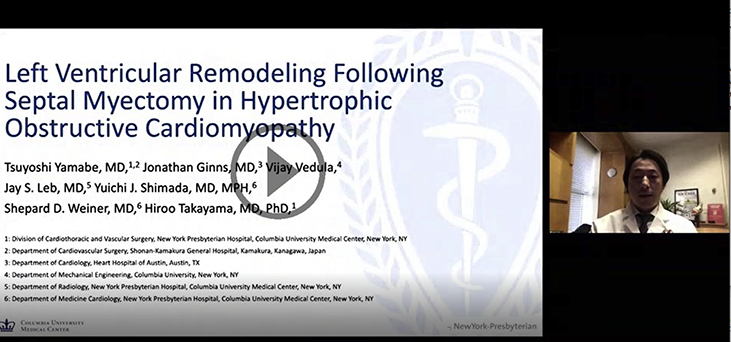

### Conflict of Interest Statement

The authors reported no conflicts of interest.

The *Journal* policy requires editors and reviewers to disclose conflicts of interest and to decline handling or reviewing manuscripts for which they may have a conflict of interest. The editors and reviewers of this article have no conflicts of interest.
